# Distribution of virulence-associated genes and antimicrobial susceptibility in clinical *Acinetobacter baumannii* isolates

**DOI:** 10.18632/oncotarget.24651

**Published:** 2018-04-24

**Authors:** Chao Liu, Yaowen Chang, Ying Xu, Yun Luo, Linrong Wu, Zhanjun Mei, Shigang Li, Rui Wang, Xu Jia

**Affiliations:** ^1^ Non-coding RNA and Drug Discovery Key Laboratory of Sichuan Province, Chengdu Medical College, Chengdu, Sichuan, China; ^2^ Clinical Laboratory, The First Affiliated Hospital of Chengdu Medical College, Chengdu, Sichuan, China

**Keywords:** Acinetobacter baumannii, antimicrobial resistance, MDR, virulence genes, PFGE

## Abstract

*Acinetobacter baumannii* is undoubtedly one of the most clinically significant pathogens. The multidrug resistance and virulence potential of *A. baumannii* are responsible for hospital-acquired nosocomial infections. Unlike numerous investigations on the drug-resistant epidemiology of *A. baumanni*, virulence molecular epidemiology is less studied. Here, we collected 88 *A. baumannii* clinical isolates, tested their antimicrobial susceptibility to 10 commonly used antibiotics and analyzed the distribution of 9 selected virulence-associated genes, aims to investigate the primary characteristics of the virulence-associated genes that exist in clinically multidrug resistant (MDR) and non-MDR isolates of *A. baumannii*. The MIC results showed the resistance rates of ciprofloxacin (68.2%, 60/88), gentamicin (67.0%, 59/88), amikacin (58.0%, 51/88), tobramycin (58.0%, 51/88), doxycycline (67.0%, 59/88), meropenem (54.5%, 48/88) and imipenem (65.9%, 58/88) were all above 50%, except for levofloxacin (34.1%, 30/88), minocycline (1.1%, 1/88) and polymyxin B (0%, 0/88). The Pulsed field gel electrophoresis (PFGE) analysis revealed that the resistance rate of MDR *A. baumannii* isolates in the Epidemic group was predominant (79.5%, 44/58), but in the Sporadic group was only 6.7% (2/30). Further investigation on the distribution of virulence genes showed the virulence genes *bap* (95.5%), *surA1* (92.0%), *BasD* (92.0%), *paaE* (88.6%), *pld* (87.5%), *BauA* (62.5%)*, omp33-36* (59.1%) and *pglC* (53.4%) were accounted for high proportion, except for *traT* (0%). Overall, our results revealed that MDR isolates predominated in the Epidemic *A. baumannii* isolates, and contained a very high proportion of virulence genes, which may lead to high risk, high pathogenicity and high treatment challenge.

## INTRODUCTION

*Acinetobacter* spp. were glucose-non-fermentative, non-motile, non-fastidious, catalase-positive, oxidative-negative, and aerobic Gram-negative coccobacilli. *Acinetobacter baumannii* is the most clinically significant *Acinetobacter* species associated with hospital-acquired infections worldwide [1]. Although *A. baumannii* used to be considered as a low virulence pathogen, pneumonia has been the main manifestation of nosocomial infections caused by this pathogen, resulting in a significant impact on the mortality rate of patients [2]. This microorganism has also been identified as the etiological agent responsible for a wide range of other infections, including septicemia, meningitis, and more recently, severe and deadly cases of necrotizing fasciitis [3].

*A. baumannii* was originally recognized as one of the six ESKAPE pathogens (Enterococcus faecium, Staphylococcus aureus, Klebsiella pneumoniae, Acinetobacter baumannii, Pseudomonas aeruginosa, and *Enterobacter species*) by Infectious Diseases Society of America (IDSA) in 2004 [4]. Soon afterwards it quickly developed into pan-drug resistance and has since received rapid recognition as one of the most important bacterial pathogens for healthcare-associated infections (HAIs) [5, 6]. The increased multidrug resistance and long period nosocomial persistence of *A. baumannii* resulted in a serious threat to hospitalized patients. The hallmark of the extreme drug resistance (XDR) phenotype is carbapenems resistance (CR), accounting for the majority of *A. baumannii* strains in many hospitals today. CR strains are often resistant to all other routinely tested antibiotics, except polymyxins, tigecycline, and sometimes aminoglycoside [7–10]. Treatment of CR-*A. baumannii* infection therefore involves the use of combinations of last resort agents such as colistin, but their efficacy and safety are yet to be defined.

Despite the increasing importance of MDR *A. baumannii* in nosocomial infections, the role and mechanism of virulence factors in *A. baumannii* pathogenesis for human infections remain largely obscure. Recently, animal models of disease and cell infestation combined with bacterial mutagenesis have provided some valuable insights into mechanisms of *A. baumannii* pathogenesis. Some potential virulence factors seem to be important for disease, including outer membrane porins, surface structures including capsule and lipopolysaccharide, enzymes such as phospholipase D, iron acquisition systems, and regulatory proteins.

These virulence factors may involve in the infection process, such as transmission, binding to host structures, cellular damage and *A. baumannii* invasion [11]. The propensity for biofilm formation is likely to contribute to transmission, which is the initial step in disease. Established factors that contribute to *A. baumannii* biofilm formation include pili (aromatic compounds, *paaE* [12]), outer membrane proteins (surface antigen protein 1, *surA1* [13]), glycoproteins and capsular polysaccharides (O-pentasaccharide, *pglC* or *pglL* [14, 15]), and extracellular polysaccharide (Phospholipase D, *pld* [16, 17]). Binding to host structures may be the first stage in the development of pneumonia. The molecular basis for such interactions is being unraveled; adhesins that mediate binding to host cells include OmpA, Bap, and Omp33–36 [18]. Following binding to host cells is cellular damage and *A. baumannii* invasion. Once inside host cells, *BasD* and *BauA* (involved in the synthesis and transport of small, iron chelating molecules called siderophores) are required for survival [3]. Bloodstream infection is a common complication of *A. baumannii* infection; the virulence gene *traT*, encoded the R6-5 plasmid-specified outer membrane protein, has previously been shown to mediate resistance to Gram-negative bacterial killing by serum [19].

Our previous surveys revealed that the antibiotic resistance among *A. baumannii* clinical isolates is very serious. [20, 21]. And antimicrobial- resistant *A. baumannii* has very high potential to spread among ill patients in ICU (intensive care units), so it is crucial to adopt early recognition and timely implementation of appropriate infection control measures in preventing outbreaks. Owing to the lack of new antimicrobials in the pipeline for problematic MDR organisms, understanding the role and mechanism of virulence factors in *A. baumannii* pathogenesis constitute novel therapeutic targets for rational drug design. We conducted a prospective exploratory study by investigating the prevalence of *A. baumannii* within a general hospital in western China, then analyzing the distribution of 9 virulence genes, and trying to explore the primary characteristics of the virulence genes within clinically MDR and non-MDR isolates of *A. baumannii*.

## RESULTS

### Clinical information of the isolates

**Figure 1:**
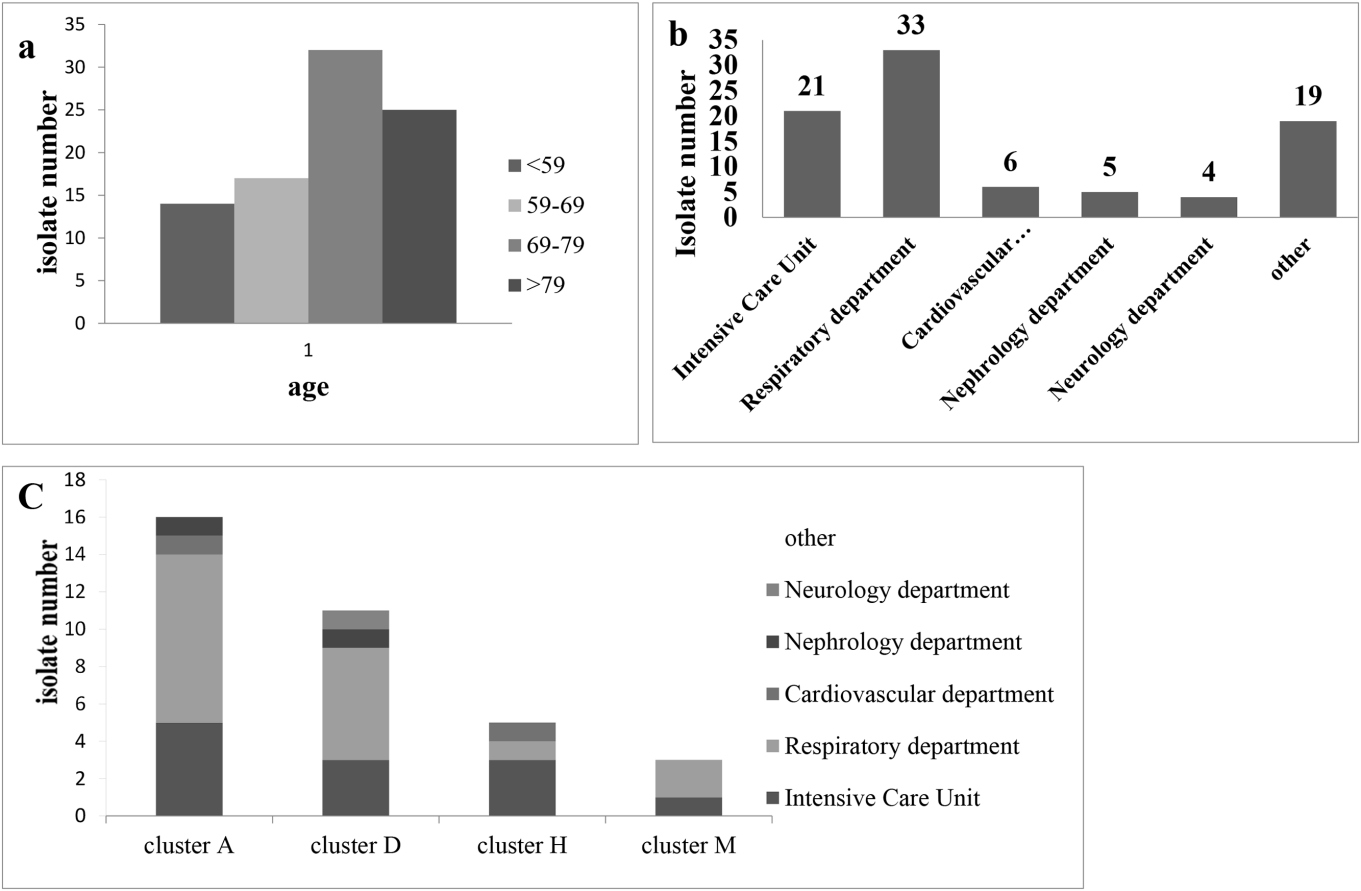
**(a)** Distribution of the age of biocides for 88 clinical isolates of *A. baumannii.* **(b)** The source distribution for 88 clinical of *A. baumannii.* **(c)** Distribution of the source for the larger cluster (A, D, H and M).

To investigate the epidemiology of *A. baumannii*, a total of 88 *A. baumannii* clinical isolates causing nosocomial infections were identified. The clinical characteristics of the 88 patients with *A. baumannii* infections were summarized in Supplementary Table 1. Epidemiological analysis of the 88 patients carrying *A. baumannii* (56 males and 32 females) revealed that 25 were ≥79 years old, 32 were between 69 and 79 years old, 17 were between 59 and 69 years old and 14 were ≤59 years old (Figure 1a). This indicates that the risk of *A. baumannii* infection would be increased with compromised immunity in aging demographics. The 88 *A. baumannii* clinical isolates were obtained from different types of specimens including sputum (71.6%, 63/88), sputum suction (13.6%, 12/88), douche (12.5%, 11/88), urine (1%, 1/88) and blood (1%, 1/88). Most of the clinical specimens were isolated from sputum and sputum suction, which is consistent with that *A. baumannii* is a major pathogen associated with respiratory tract infection. The most prevalent department of isolation was the Respiratory department (37.5%, 33/88), followed by ICU (23.9%, 21/88), Neurology department (7.9%, 7/88), Cardiovascular department (6.8%, 6/88), Nephrology department (5.7%, 5/88), and other wards (18.2%, 16/88) (Figure 1b).

### Genotyping of *A. baumannii* clinical isolates

To determine the spread of *A. baumannii* within the hospital setting, all isolates were genotyped using PFGE (Pulsed Field Gel Electrophoresis). PFGE results were analyzed by BioNumerics and pulsotype designation was based on isolates showing ≥80% relatedness. PFGE fingerprinting analysis revealed that 58 *A. baumannii* isolates (all ≥80% relatedness) were Epidemic group and the remaining 31 *A. baumannii* isolates (all <80% relatedness) were Sporadic group (Supplementary Figure 1).

The Epidemic group were classified into 13 major clusters from A to M, in which the Larger Clusters (cluster A, D, H and M, N ≥5) contains a total of 44.3%. Cluster A (19.3%, 17/88) was most frequently detected in the clinical isolates and was responsible for an epidemic between January and February in 2014, and in the Respiratory department (N = 9), ICU (N = 5), Internal Medicine Ward (N = 1), General surgery ward (N = 1) and Cardiovascular department (N = 1). Cluster D (12.5%, 11/88) was second most frequent that detected in the clinical isolates and was closely correlated in August (N = 3), July (N = 1) and September (N = 1) in 2014 and January (N = 3), August (N = 2) and July (N = 1) in 2015, with a source of Respiratory department (N = 6), ICU (N = 3), Nephrology department (N = 1) and Neurology department (N = 1). Cluster H (6.8%, 6/88) was closely correlated with April (N = 5) and July (N = 1) in 2014, and comes from the ICU (N = 3), Respiratory department (N = 1), Geriatric Medicine (N = 1) and Cardiovascular department (N = 1). Cluster M (5.7%, N = 5) was closely correlated with December in 2013 (N = 4) and August in 2014, and distributed in the Respiratory department (N = 2), ICU (N = 2) and Digestive System Department (N = 1) (Supplementary Table 1). Although a small proportion of these Larger Clusters were discretely distributed at different wards, most of the strains were found within were mainly concentrated in respiratory and ICU wards (Figure 1c).

### Antimicrobial susceptibility of *A. baumannii* clinical isolates

To understand the drug-resistance of 88 *A. baumannii* clinical isolates, antimicrobial susceptibility testing was performed. The results showed that the resistance rates to levofloxacin, ciprofloxacin, gentamicin, amikacin, tobramycin, minocycline, doxycycline, meropenem, imipenem and polymyxin B accounted for 34.1% (30/88), 68.2% (60/88), 67.0% (59/88), 58.0% (51/88), 58.0% (51/88), 1.1% (1/88), 67.0% (59/88), 54.5% (48/88), 65.9% (58/88) and 0% (0/88), respectively (Table 1). The resistance rates of ciprofloxacin, gentamicin, amikacin, tobramycin, doxycycline, meropenem and imipenem were all above 50%, while those of levofloxacin, polymyxin B and minocycline were only 34.1%, 0% and 1.1%, respectively. Most importantly, in the Larger Clusters (cluster A, D, H and M), the rates of resistance to some antibiotics were almost over 80% (Table 1). From these results we can reasonably infer that minocycline and polymyxin B may be used as empiric treatment agents for *A. baumannnii* in China. Interestingly, the resistance rate of all the antibiotics in the Epidemic group was significantly higher than that of the Sporadic group, except for minocycline and polymyxin B (P value <0.05).

**Table 1:** Data of *A. baumannii* isolates resistant to selected antibiotics and genotyping

Antimicrobial agents	All	Larger Clusters (N=39)				Epidemic	Sporadic	P value
	88 (%)	Cluster A	Cluster D	Cluster H	Cluster M	58 (n, %)	30 (n, %)	
		17 (%)	11 (%)	6 (%)	5 (%)			
LEV	34.1	58.8	45.5	33.3	60.0	28, 48.3	2, 6.7	<0.000
CIP	68.2	94.1	90.9	100	80.0	51, 87.9	9, 30	<0.000
MIN	1.1	0	0	0	20.0	1, 1.7	0	1
DOX	67.0	100	90.9	100	80.0	51, 87.9	8, 26.7	<0.000
AMK	58.0	100	54.5	100	60.0	47, 81.0	4, 13.3	<0.000
GEN	67.0	100	63.6	100	80.0	49, 84.5	10, 33.3	<0.000
TOB	58.0	94.1	54.5	100	60.0	45, 77.6	6, 20	<0.000
MERO	54.5	94.1	72.7	100	60.0	43, 74.1	5, 16.7	<0.000
IMP	65.9	100	90.9	100	60.0	50, 86.2	8, 26.7	<0.000
PB	0	0	0	0	0	0	0	
MDR	52.3	94.1	54.5	100	60.0	44, 75.9	2, 6.7	<0.000

LEV, Levofloxacin; CIP, Ciprofloxacin; MIN, Minocycline; DOX, Doxycycline; AMK, Amikacin; GEN, Gentamicin; TOB, Tobramycin; IMP, Imipenem; MERO, Meropenem; Polymyxin B, PB; MDR, Multidrug resistance; P value, Chi-square test or Fisher exact test. Larger Clusters mean clusters contains a larger number of strains (N≥5).

For cross-resistance profile in these isolates, we found that the prevalence of MDR *A. baumannii* isolates was 52.3% (46/88) in the present study. Combined with the genotype data of these 88 *A. baumannii* isolates, the resistance rate of MDR *A. baumannii* in the Sporadic group was only 6.7% (2/30) (P value <0.01, Table 1). On the contrary, MDR *A. baumannii* isolates almost predominated in the Epidemic group, and *A. baumannii* isolates in cluster A (94.1%, 16/17) and cluster H (100%, 6/6) were almost all MDR. Above all, the high proportion of MDR *A. baumannii* in the Epidemic group indicated that MDR *A. baumannii* is more likely to spread and cause infection in the hospital.

### Prevalence of virulence genes among *A. baumannii*

To investigate the virulence potential of 88 *A. baumannii* clinical isolates, PCR was conducted to detected 9 virulence associated genes (mentioned in the Introduction section and listed in Table 2). The results showed that the virulence genes *bap* (95.5%, 84/88), *surA1* (92.0%, 81/88), *BasD* (92.0%, 81/88), *paaE* (88.6%, 78/88) and *pld* (87.5%, 77/88) were accounted for high proportion (>80%) in these 88 *A. baumannii* isolates. Meanwhile, more than half of the clinical isolates carried *BauA* (62.5%, 55/88)*, omp33-36* (59.1%, 52/88) and *pglC* (53.4%, 47/88). However, no *traT* (serum resistance gene) were detected in these *A. baumannii* isolates (Table 3).

**Table 2:** Virulence genes among multidrug resistance and genotyping *Acinetobacter baumannii* strains isolated

	All (n, %)	cluster A (N=17)	cluster D (N=11)	cluster H (N=6)	cluster M (N=5)	MDR (N=46) (n, %)	non- MDR (N=42) (n, %)	P value	Epidemic N=58 (n, %)	Sporadic N=30 (n, %)	P value
*bap*	84, 95.5	100	100	100	80.0	45, 97.8	39, 92.9	0.545	57, 98.3	27, 90.0	0.22
*surA1*	81, 92.0	100	100	100	60.0	46, 100	35, 83.3	0.004	55, 94.5	26, 86.8	0.355
*BasD*	81, 92.0	100	100	100	60.0	44, 95.7	37, 88.1	0.361	55, 94.5	26, 86.9	0.071
*BauA*	55, 62.5	88.2	72.7	83.3	60.0	36, 78.3	19, 45.2	0.001	44, 75.9	11, 36.7	<0.000
*pld*	77, 87.5	100	100	100	40.0	44, 95.7	33, 78.6	0.016	53, 91.4	24, 80	0.234
*omp-33-36*	52, 59.1	52.9	63.6	66.7	40.0	26, 56.5	26, 61.9	0.608	29, 50.0	23, 76.7	0.016
*paaE*	78, 88.6	100	90.9	100	60.0	45, 97.8	33, 78.6	0.012	54, 93.1	24, 80	0.138
*pglC*	47, 53.4	41.2	72.7	16.7	60.0	21, 45.7	26, 61.9	0.127	31, 53.4	16, 53.3	0.992
*tra T*	0	0	0	0	0	0	0		0	0	

**Table 3:** Virulence genes among antibiotic resistance and sensitive of *Acinetobacter baumannii* strains isolated

Virulence genes	LEV		P	CIP		P	MIN		P	DOX		P	AMK		P	GEN		P	TOB		P	IMP		P	MERO		p
	R 30	Non-R 58		R 60	Non-R 28		R 1	Non-R 87		R 59	Non-R 29		R 51	Non-R 37		R 59	Non-R 29		R 51	Non-R 37		R 58	Non-R 30		R 48	Non-R 40	
*bap*	29	55		58	26		1	26		57	27		50	34		57	27		49	35		57	27		47	37	
*surA1*	28	53		58	23		0	23		57	24	^*^	51	30	^*^	57	24		50	31	^*^	58	23	^*^	48	33	^*^
*BasD*	27	54		56	25		0	25		55	26		49	32		55	26		48	33		56	25		46	35	
*BauA*	25	30	^*^	46	9	^*^	0	8^*^	^*^	46	9	^*^	41	14	^*^	45	10	^*^	40	15	^*^	44	11	^*^	36	19	^*^
*pld*	26	51		56	21	^*^	0	21		55	22	^*^	49	28	^*^	55	22	^*^	49	28	^*^	56	21	^*^	46	31	^*^
*omp33-36*	12	40	^*^	35	17		0	18		33	19		29	23		34	18		30	22		35	17		31	21	
*paaE*	28	50		57	21	^*^	0	21		56	22	^*^	50	28	^*^	55	23		49	29	^*^	56	22	^*^	47	31	
*pglC*	15	32		31	16		1	16		31	16		24	23		28	19		23	24		29	18		22	25	
*tra T*	0	0		0	0		0	0		0	0		0	0		0	0		0	0		0	0		0	0	

LEV, Levofloxacin; CIP, Ciprofloxacin; MIN, Minocycline; DOX, Doxycycline; AMK, Amikacin; GEN, Gentamicin; TOB, Tobramycin; IMP, Imipenem; MERO, Meropenem; R, Resistant; non-R, non- resistant; P, P value, Chi-square test or Fisher exact test; ^*^, P value < 0.05; Same as in Table 2.

Associated with the drug-resistance of 88 *A. baumannii* clinical isolates, the results revealed that the distribution of each virulence associated gene has some unique characteristics. The rate of *surA1* among aminoglycosides (tobramycin and amikacin), doxycycline and carbapenems (imipenem and meropenem) resistant isolates was significantly higher than that in homologous sensitive isolates. The rate of *BauA* among the vast majority of antibiotics resistant (levofloxacin, ciprofloxacin, gentamicin, tobramycin, amikacin, doxycycline, imipenem and meropenem) isolates was significantly higher than that of homologous sensitive isolates. The rate of *pld* among doxycycline, aminoglycosides and carbapenem resistant isolates was significantly higher than that of homologous sensitive isolates. The rate of *paaE* among ciprofloxacin, gentamicin, tobramycin, amikacin, doxycycline, imipenem and meropenem resistant isolates was significantly higher than that of homologous sensitive isolates. But what’s interesting is that the rate of *omp33-36* among levofloxacin sensitive isolates was significantly higher than that of levofloxacin resistance isolates. In summary, these relationships are statistically significant (P value <0.05) (Table 4), and the antibiotic-resistant *A. baumannii* carries more virulence genes.

**Table 4:** Virulence genes primer used in this study and the protein functions

Virulence gene	Nucleotide sequence	Protein function/description	Reference
*bap*	F: AGTTAAAGAAGGGCAAGAAG R: GGAGCACCACCTAACTGA	Biofilm maturation, maintenance	[54]
*surA1*	F: CAATTGGTAGCTGGCGATCA R: TTAGGCGGGACTCAGCTTTT	Surface antigen protein 1	[13]
*BasD*	F: CTCTTGCATGGCAACACCAC R: CCAACGAGACCGCTTATGGT	Acinetobactin biosynthesis Iron acquisition system	[55, 56]
*BauA*	F: TGGCAAGGTGAAAATGCACG R: GCCGCATATGCCATCAACTG	Acinetobactin transport Iron acquisition system	
*Pld*	F: CCGTCAATTACGCCAAGCTG R: CTGACGCTACCTGACGGTTT	Phospholipase D	[57, 58]
*omp33-36*	F: ATTAGCCATGACCGGTGCTC R: CCACCCCAAACATGGTCGTA	Outer membrane porin	[59–61]
*paaE*	F: CTATTTAGGCGTTGCTGCGG R: CCTTACAACGACAGGTCGCA	phenylalanine catabolic pathway	[62, 63]
*pglC*	F: TGGATGAGTTAGCTGC R: TTTTACAAATAGTTAAGC	O-glycosylation system capsular polysaccharide	[14, 64]
*traT*	F: GGTGTGGTGCGATGAGCACAG R: CACGGTTCAGCCATCCCTGAG	Serum-resistance-associated	[65]

Further analysis in cross-resistance profile found that, the carriage ratio of *surA1, BauA*, *pld* and *paaE* in MDR *A. baumannii* isolates (100.0%, 78.3%, 95.7%, 97.8%) was significantly higher (P value <0.05) than in non-MDR *A. baumannii* isolates (83.3%, 45.2%, 78.6%, 78.6%), and the carriage ratio of *BasD* in Epidemic isolates (94.5%) was significantly higher (P value <0.05) than in Sporadic isolates (86.9%) (Table 3).

## DISCUSSION

*Acinetobacter* spp. was initially considered as an opportunistic human pathogen of low virulence with minimal significance. However, during the last two decades, the increasing ubiquity and intensity of mechanical ventilation, central venous and urinary catheterization, and antibacterial therapy have caused a surge in the frequency and severity of *Acinetobacter* infections [22–24]. *A. baumannii* is endowed with an open pan genome, which means it can easily acquire new function. The persistence of *A. baumannii* in the hospital setting, together with the strong selection pressure imposed by the use of antimicrobials in the clinical practice, has promoted the evolution of clinical *A. baumannii* towards drug resistance isolates and the formation of some virulence factors which facilitate the survival of *A. baumannii* in the hospital [25].

Drug-resistant *A. baumannii* is more likely to survive in hospital environment, causing an outbreak in the hospital [26, 27]. The treatment of *A. baumannii* infections is becoming a serious problem because of its strong antibiotic resistance [28]. Especially, surveillance by the CHINET project demonstrated that resistance of *A. baumannii* strains to carbapenems increased from 31 to 66.7% between 2005 and 2014 [29]. The current data showed that the resistance rate of imipenem (65.9%, Table 1) was higher than our previous report (60.9%) [10]. Although the resistance rate is lower than in Turkey (94.9%) [30] and Iran (82%) [31], it is still in a rising tendency. This suggests that carbapenems can’t be habitually used in *A. baumannii* infectious treatment.

Here, we found that the MDR *A. baumannii* in the Epidemic group (44/58, 75.9%) was higher than that of Sporadic group (2/30, 6.7%) (P value < 0.05, Table 1). In addition, the Larger Clusters A, D, H and M in the Epidemic group are all substantially composed of MDR *A. baumannii*, and showed resistance to most of the antibiotics. This result may due to the clinical abuse of antibiotics, and resistant *A. baumanii* was screened to survive, and then gradually developed into MDR *A. baumannii*. So, their persistent selection and ongoing transmission in hospital may result in epidemic outbreak [32, 33].

Traditional thinking was that antibiotic resistance caused a metabolic cost to the bacterium and hence the “anti-virulence” factor [34]. However, the White paper reported that a lung and blood clinical isolate (*A. baumannii* HUMC1) is not only an extreme drug-resistant isolate (almost resistant to all antibiotics, except colistin), but also a hyper-virulent isolate [35]. Meanwhile, some researchers have also suggested that MDR *A. baumannii* can survive proliferation even after the host treated with antibiotics, and then producing a variety of metabolites that upset the physiological balance of the host as a virulence effect [13, 36]. Accordingly, the clinical outcome of *A. baumannii* infections is jointly influenced by antimicrobial resistance and virulence factor.

The infection symptoms caused by *A. baumannii* are closely associated with its virulence factor. But the virulence genes distribution in clinical *A*. *baumannii* were rarely reported, except for *bap, omp33-36* and *traT* [37, 38]. This work examined the distribution of 9 virulence genes in clinically collected *A. baumannii* isolates. Our results showed that the existence rate of each virulence gene in the *A. baumannii* isolates was more than 50%, except *traT* (Table 3). Furthermore, the virulence genes carriage rate of *A. baumannii* in MDR was significantly higher than that of non-MDR *A. baumannii* (P< 0.05, Table 3), such as *surA1, BauA, pld* and *paaE*. This suggests that the drug resistant *A. baumannii* isolates appear to have greater potentials for toxicity.

Biofilm is an important virulence factor of *A. baumannii*. Loehfelm et al. suggested the development and thickness of the mature biofilm structure and intercellular adhesion were closely associated with Bap family proteins (coded gene *bap*) [39]. The *bap* genes were detected with very high frequency in clinical *A. baumannii* from Iran (92%) [40], Republic of Korea (100%) [37] and USA (84%) [41]. This work also showed that *A. baumannii* isolates frequently possess *bap* genes (95.5%). These results indicate that most *A. baumannii* may have the biofilm formation ability.

Baumannii acinetobactin utilization (BauA) is an outer membrane protein, acting out the siderophore-ferric complex receptor [42]. Whole protein includes 2 domains comprising a cork domain at N terminal of the protein and a transmembrane barrel at the C terminal [43]. This work showed the rate of virulence gene *BauA* was significantly higher in drug-resistant *A. baumannii* (P<0.05, Table 4), and *A. baumannii* isolates with *BauA* gene seems to show a wide range of drug resistance. So, we speculate that the transmembrane barrel may be associated with drug resistance in *A. baumannii*.

*A. baumannii* phenylacetate degradation pathway was a hybrid pathway [12], and a key step in this pathway is performed by the *paaE* gene [44]. This study indicates that the rates of *paaE* were higher in the ciprofloxacin, tobramycin, amikacin, doxycycline or imipenem resistant *A. baumannii* isolates than in the corresponding sensitive isolates (P< 0.05, Table 4). We speculate these antibiotics may be metabolized as substrates during the metabolism of phenylacetic.

Some recent studies have reported that the virulence gene *omp33-36* contributes to resistance to carbapenems in *A. baumannii* [45–47]. But in our work, there was no significant difference in the frequency of *omp33-36* gene carried between carbapenems-resistant and -sensitive *A. baumannii* (Table 4). In contrast, our work showed that *A. baumannii*, which was found to be levofloxacin-sensitive, appears to carry more virulence gene *omp33-36*, and only a few *A. baumannii* isolates developed resistance to levofloxacin. We think levofloxacin is an effective drug for the treatment of *A. baumannii* infection.

A recent study reported that *traT* (80%) was found high frequency in CR-*A. baumannii* isolates in Iran [38]. However, this study showed that none of the 88 *A. baumannii* clinical isolates carried the *traT* gene, which was similar to the report of Brasil, 2004 [48]. This indicates that *traT* gene frequently existing in CR- *A. baumannii* is not a universal phenomenon, for the moment only finding in Iran.

In summary, our findings showed that the prevalence of MDR *A. baumannii* isolates collected from different wards was 52.3%, and the MDR *A. baumannii* in the Epidemic group was significant higher than that of Sporadic group. Virulence associated genes detected in this study accounted for a high proportion among the 88 *A. baumannii* clinical isolates, especially in the MDR *A. baumannii* isolates, which means high risk, high pathogenicity and high treatment challenge. At the same time, our work indicates that some potential links between virulence genes and drug resistance. This work strongly suggests that hospitals need to strengthen infection control to prevent *A. baumannii* from causing an outbreak of infection.

## MATERIALS AND METHODS

### Bacterial isolates and growth conditions

The 88 nonrecurring clinical isolates of *A. baumannii* used in this study were collected from patients with nosocomial infections during the period from April 2013 to October 2015, at the First Affiliated Teaching Hospitals of Chengdu Medical College in Sichuan province, China. All isolates were collected as part of routine care, data were collected, identified and handled anonymously. The strains were stored in glycerol at -80°C and bacteria were grown on MacConkey Agar (MCA plates) (Oxoid, England) before tested. Isolates were identified by VITEK 2 (bioMe’rieux, Marcy-l’E’toile, France) automated microbiology system. Besides, all isolates were further verified by detecting the existence of the *rpoB* and sequencing the *16S-23S rRNA* gene spacer region [10].

### Pulsed-field gel electrophoresis testing

All the 88 *A. baumannii* isolates were genotyped by using Pulsed-Field Gel Electrophoresis (PFGE) analysis as previously described [49]. *A. baumannii* isolates were grown on Mueller-Hinton agar medium plates overnight at 37 °C and bacterial genomic DNAs were prepared. After the genomic DNA was digested with ApaI (TaKaRa, Dalian, China), the DNA fragments were separated by electrophoresis in 1% gold agarose (Lonza) in 0.5 × Tris - borate - EDTA buffer with a CHEF apparatus (CHEF Mapper XA, Bio-Rad, USA). The conditions included 14°C and 6V/cm with alternating pulses at a 120° angle with a 5-35s pulse time gradient for a total of 22 h. The same method was also used to extract the *Salmonella enterica* serotype Braenderup H9812 genomic DNAs, which was used as the size marker after being digested by *Xba*I (TaKaRa, Dalian, China) [50]. The similarity examination of DNA patterns analyzed with BioNumerics 7.0 (Applied Math, USA) was based on Tenover criteria and Dice coefficient, and 80% relatedness was used as the threshold to distinguish the pulsotype [51].

### Antimicrobial susceptibility testing

The 88 *A. baumannii* strains were tested for their susceptibilities to 10 antibiotics: levofloxacin, ciprofloxacin, gentamicin, amikacin, tobramycin, minocycline, doxycycline, meropenem, imipenem and polymyxin B. The minimal inhibitory concentrations (MICs) were determined by agar dilution method and interpreted as previously described in the guidelines according to the 2017 Clinical and Laboratory Standards Institute (CLSI) [52]. *Escherichia coli* ATCC 25922 and *Pseudomonas aeruginosa* ATCC 27853 were both used as quality control strains. The MICs value used to indicate antibiotic resistance are ≥4 *μg/ml* for ciprofloxacin and polymyxin B in accordance with CLSI, ≥8 *μg/ml* for levofloxacin, meropenem and imipenem, ≥16 *μg/ml* for gentamicin, tobramycin, minocycline and doxycycline, and ≥64 *μg/ml* for amikacin.

### Definition of multidrug resistance (MDR)

Base on researchers’ definition of MDR among *A. baumannii* in many published works [53], we defined MDR *A. baumannii* as containing resistance to any combination of 3 or more of the 4 drug classes commonly used to treat Gram negative infections: fluoroquinolones, aminoglycosides, carbapenems and tetracyclines. Resistance to an antibiotic class was defined as resistance to all drugs representative of that class in this panel.

### PCR amplification and DNA sequencing for virulence genes detecting

9 virulence genes as listed in Table 2 were detected by PCR to investigate the distribution in the 88 *A. baumannii* strains. The primers used were also listed in Table 2 and PCR was carried out according to references in the same table. The PCR products were sequenced by TSINGKE (Chengdu, China) and then confirmed by sequence alignment to the corresponding genes of *A. baumannii* with NCBI Nucleotide Blast (http://blast.ncbi.nlm.nih.gov/Blast.cgi)

## SUPPLEMENTARY MATERIALS FIGURE AND TABLE




