# Supplementary material for: Distribution of virulence-associated genes and antimicrobial susceptibility in clinical Acinetobacter baumannii isolates
Source: Oncotarget. 2018 Apr 24;9(31):21663–73. doi: 10.18632/oncotarget.24651 (PMC5955172; doi:10.18632/oncotarget.24651)
Supplement: Supplementary file 1 [file oncotarget-09-21663-s001.pdf]

Distribution of virulence-associated genes and antimicrobial susceptibility in clinical *Acinetobacter baumannii* isolates

SUPPLEMENTARY MATERIALS

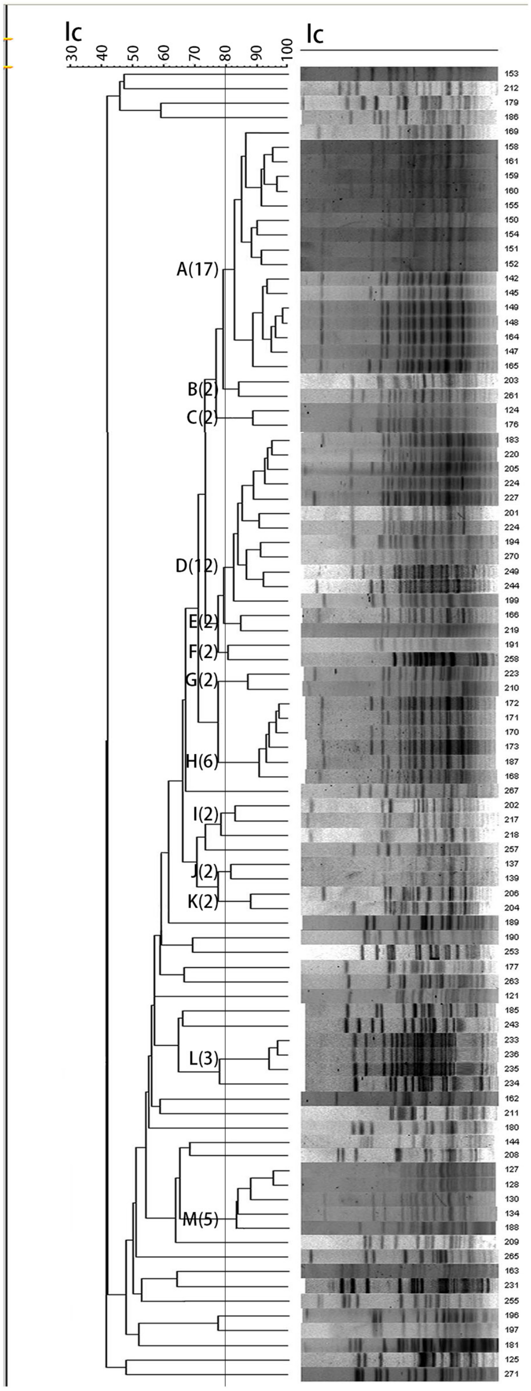

Supplementary Figure 1: A dendrogram of PFGE. All the 88 *A. baumannii* isolates were classified into epidemic (cluster A to M) and Sporadic. PFGE, pulsed field gel electrophoresis; A to M, cluster A to M.

Supplementary Table 1: The clinical characteristics of 88 strains of *Acinetobacter baumannii*

See Supplementary File 1
