# Supplementary material for: Distribution of virulence-associated genes and antimicrobial susceptibility in clinical Acinetobacter baumannii isolates
Source: Oncotarget. 2018 Apr 24;9(31):21663–73. doi: 10.18632/oncotarget.24651 (PMC5955172; doi:10.18632/oncotarget.24651)
Supplement: Supplementary file 2 [file oncotarget-09-21663-s002.docx]

**Supplementary Table 1: The clinical characteristics of 88 strains of *Acinetobacter baumannii***

| ID | PFGE (80%) | Age | Gender | Source | Department | Sampling time |
| --- | --- | --- | --- | --- | --- | --- |
| 206 | cluster K | 73 | male | sputum | Respiratory department | 2013.04 |
| 204 | cluster K | 78 | male | sputum suction | Respiratory department | 2013.10 |
| 121 |  | 51 | male | sputum suction | Intensive Care Unit | 2013.11 |
| 127 | cluster M | 71 | male | sputum | Respiratory department | 2013.12 |
| 128 | cluster M | 81 | male | douche | Respiratory department | 2013.12 |
| 130 | cluster M | 76 | male | sputum | Internal Medicine Ward | 2013.12 |
| 134 | cluster M | 78 | female | sputum suction | Intensive Care Unit | 2013.12 |
| 124 | cluster C | 86 | male | sputum suction | Intensive Care Unit | 2013.11 |
| 125 |  | 70 | female | sputum | Cardiovascular department | 2013.11 |
| 142 | cluster A | 59 | male | sputum suction | Intensive Care Unit | 2014.01 |
| 137 | cluster J | 80 | male | sputum | Respiratory department | 2014.01 |
| 145 | cluster A | 77 | female | sputum suction | Respiratory department | 2014.01 |
| 147 | cluster A | 78 | male | douche | Respiratory department | 2014.01 |
| 148 | cluster A | 76 | female | sputum suction | Intensive Care Unit | 2014.01 |
| 149 | cluster A | 79 | female | sputum suction | Intensive Care Unit | 2014.02 |
| 150 | cluster A | 45 | male | sputum | General surgery ward | 2014.02 |
| 151 | cluster A | 85 | male | sputum | Respiratory department | 2014.02 |
| 152 | cluster A | 79 | male | sputum | Respiratory department | 2014.02 |
| 139 | cluster J | 81 | male | sputum | Respiratory department | 2014.01 |
| 154 | cluster A | 79 | male | douche | Respiratory department | 2014.02 |
| 155 | cluster A | 79 | female | douche | Respiratory department | 2014.02 |
| 158 | cluster A | 81 | male | sputum suction | Intensive Care Unit | 2014.02 |
| 159 | cluster A | 78 | male | sputum | Internal Medicine Ward | 2014.02 |
| 160 | cluster A | 77 | male | sputum | Respiratory department | 2014.02 |
| 161 | cluster A | 69 | female | douche | Respiratory department | 2014.03 |
| 144 |  | 31 | male | sputum | Nephrology department | 2014.01 |
| 153 |  | 60 | female | sputum | NA | 2014.02 |
| 164 | cluster A | 68 | male | sputum | Cardiovascular department | 2014.03 |
| 165 | cluster A | 81 | male | sputum | Respiratory department | 2014.04 |
| 162 |  | 77 | female | sputum | Digestive System Department | 2014.03 |
| 168 | cluster H | 80 | female | sputum suction | Intensive Care Unit | 2014.04 |
| 169 | cluster A | 80 | female | sputum suction | Intensive Care Unit | 2014.04 |
| 170 | cluster H | 45 | male | sputum | Geriatric Medicine | 2014.04 |
| 171 | cluster H | 62 | male | sputum suction | Intensive Care Unit | 2014.04 |
| 172 | cluster H | 74 | female | sputum | Cardiovascular department | 2014.04 |
| 173 | cluster H | 66 | male | sputum | Respiratory department | 2014.04 |
| 163 |  | 94 | male | sputum | Respiratory department | 2014.03 |
| 166 | cluster E | 70 | male | sputum | Cardio-Thoracic Surgery | 2014.04 |
| 176 | cluster C | 62 | male | sputum | Respiratory department | 2014.05 |
| 177 |  | 62 | female | douche | Respiratory department | 2014.06 |
| 179 |  | 65 | male | sputum | Oncology Department | 2014.06 |
| 183 | cluster D | 69 | female | douche | Respiratory department | 2014.07 |
| 181 |  | 83 | male | sputum | Respiratory department | 2014.07 |
| 185 |  | 4 | female | sputum | Pediatrics department | 2014.07 |
| 187 | cluster H | 37 | female | sputum | Intensive Care Unit | 2014.07 |
| 188 | cluster M | 76 | male | sputum | Digestive System Department | 2014.08 |
| 186 |  | 72 | male | sputum | Oncology Department | 2014.07 |
| 203 | cluster B | 37 | male | sputum | Respiratory department | 2014.07 |
| 180 |  | 77 | female | sputum | Cardiovascular department | 2014.07 |
| 194 | cluster D | 68 | female | douche | Respiratory department | 2014.08 |
| 189 |  | 81 | male | sputum | Neurosurgery | 2014.08 |
| 190 |  | 82 | male | sputum | Cardiovascular department | 2014.08 |
| 199 | cluster D | 73 | male | douche | Respiratory department | 2014.08 |
| 201 | cluster D | 66 | male | sputum | Respiratory department | 2014.09 |
| 191 | cluster F | 77 | female | sputum | Cardiovascular department | 2014.08 |
| 196 |  | 80 | male | sputum | Respiratory department | 2014.08 |
| 197 |  | 73 | female | sputum | Respiratory department | 2014.08 |
| 205 | cluster D | 57 | female | sputum | Respiratory department | 2014.08 |
| 202 | cluster I | 65 | male | sputum | Infections department | 2014.09 |
| 210 | cluster G | 78 | female | sputum | Intensive Care Unit | 2015.01 |
| 208 |  | 48 | male | sputum | Intensive Care Unit | 2015.01 |
| 211 |  | 72 | female | sputum | Emergency department | 2015.01 |
| 209 |  | 51 | male | sputum | Intensive Care Unit | 2015.01 |
| 212 |  | 78 | female | sputum | Emergency department | 2015.01 |
| 217 | cluster I | 66 | male | sputum | Nephrology department | 2015.01 |
| 218 |  | 61 | female | sputum | Intensive Care Unit | 2015.01 |
| 219 | cluster E | 52 | male | sputum | Intensive Care Unit | 2015.01 |
| 220 | cluster D | 61 | female | sputum | Nephrology department | 2015.01 |
| 223 | cluster G | 52 | male | sputum | Intensive Care Unit | 2015.01 |
| 224 | cluster D | 45 | male | sputum | Intensive Care Unit | 2015.01 |
| 227 | cluster D | 77 | male | sputum | Neurology department | 2015.01 |
| 263 |  | 69 | male | douche | Respiratory department | 2015.10 |
| 257 |  | 67 | female | sputum | Endocrinology Department | 2015.6 |
| 261 | cluster B | 89 | male | sputum | Intensive Care Unit | 2015.6 |
| 253 |  | 66 | male | urine | Nephrology department | 2015.6 |
| 255 |  | 71 | female | sputum | Neurosurgery | 2015.6 |
| 258 | cluster F | 69 | male | sputum | Respiratory department | 2015.6 |
| 244 | cluster D | 89 | male | sputum | Intensive Care Unit | 2015.7 |
| 249 | cluster D | 22 | female | sputum | Intensive Care Unit | 2015.8 |
| 235 | cluster L | 90 | male | douche | Respiratory department | 2015.7 |
| 231 |  | 70 | male | sputum | Neurosurgery | 2015.7 |
| 236 | cluster L | 86 | female | sputum | Neurology department | 2015.7 |
| 243 |  | 71 | male | sputum | Neurology department | 2015.7 |
| 234 |  | 64 | female | sputum | ombination of traditional chinese medicine and western medicine | 2015.7 |
|  |  |  |  |  |  |  |
| 233 | cluster L | 81 | male | sputum | Neurology department | 2015.7 |
| 271 |  | 84 | female | sputum | Respiratory department | 2015.8 |
| 270 | cluster D | 82 | male | sputum | Respiratory department | 2015.8 |
| 265 |  | 69 | male | blood | Nephrology department | 2015.9 |

ID, isolates ID; PFGE, pulsed field gel electrophoresis; NA, not Available.
